# Design of freeform geometries in a MEMS accelerometer with a mechanical motion preamplifier based on a genetic algorithm

**DOI:** 10.1038/s41378-020-00214-1

**Published:** 2020-11-30

**Authors:** Chen Wang, Xiaoxiao Song, Weidong Fang, Fang Chen, Ioannis Zeimpekis, Yuan Wang, Aojie Quan, Jian Bai, Huafeng Liu, Gerold Schropfer, Chris Welham, Michael Kraft

**Affiliations:** 1 College of Optical Science and Engineering, Zhejiang University, Hangzhou, 310027 P.R. China; 2 Department of Electrical Engineering and Computer Science, University of Liege, Liege, B-4000 Belgium; 3 ESAT-MICAS, University of Leuven, Leuven, 3001 Belgium; 4 PGMF and School of Physics, Huazhong University of Science and Technology, Wuhan, 430074 P.R. China; 5 Shanghai Institute of Microsystem and Information Technology, Chinese Academy of Sciences, Shanghai, 200050 P.R. China; 6 ORC, University of Southampton, Southampton, SO17 1BJ UK; 7 Coventor—A Lam Research Company, Villebon sur Yvette, 91140 France

**Keywords:** Electrical and electronic engineering, Nanoscale devices

## Abstract

This paper describes a novel, semiautomated design methodology based on a genetic algorithm (GA) using freeform geometries for microelectromechanical systems (MEMS) devices. The proposed method can design MEMS devices comprising freeform geometries and optimize such MEMS devices to provide high sensitivity, large bandwidth, and large fabrication tolerances. The proposed method does not require much computation time or memory. The use of freeform geometries allows more degrees of freedom in the design process, improving the diversity and performance of MEMS devices. A MEMS accelerometer comprising a mechanical motion amplifier is presented to demonstrate the effectiveness of the design approach. Experimental results show an improvement in the product of sensitivity and bandwidth by 100% and a sensitivity improvement by 141% compared to the case of a device designed with conventional orthogonal shapes. Furthermore, excellent immunities to fabrication tolerance and parameter mismatch are achieved.

## Micro-electrical-mechanical systems: New, powerful design methodology for micro-electrical-mechanical system devices

Based on a genetic algorithm using freeform geometries, a novel, powerful, semi-automated design methodology has been devised that can be applied to a wide range of micro-electrical-mechanical system (MEMS) devices. Thanks to their small size and low cost, MEMS devices find wide application in many areas, but the conventional, simple designs of the devices often limit their performance. A team headed by Jian Bai at Zhejiang University, China and Michael Kraft at University of Leuven, Belgium, has developed a method for designing MEMS devices in which the use of freeform geometries offers greater freedom in the design process, thereby enhancing the diversity and performance of the devices. The experimental results indicated a sensitivity improvement of 141% compared with a conventionally designed device. The authors believe that their methodology can be applied to many MEMS devices and result in unconventional geometric shapes with higher performance.

## Introduction

Microelectromechanical systems (MEMS) devices are widely used in various areas due to their small size, light weight, and low cost^1^. The vast majority of MEMS devices, such as accelerometers^2^, gyroscopes^3^, pressure sensors^4^, microgrippers^5^, and microphones^6^, rely on simple geometrical layouts comprising only a few simple building blocks, such as beams, rectangular masses and, more rarely, rings or disk-shaped structures. As discussed in the following, there are cases in which such conventional, simple designs limit the performance of MEMS devices and therefore may not meet the requirements for specific applications. Unlike conventional designs, geometries with more complex structures offer the designer more freedom, which may result in novel designs with superior performance^7^ and overcome the limitation of simple mechanisms^8^. For example, Middlemiss et al.^8^ and Boom et al.^9^ improved the resolution of accelerometers to the nano-g level with curved anti-springs. These anti-springs exhibit a low effective spring constant that cannot be achieved by conventional orthogonal designs (ODs) with the same fabrication constraints. Nguyen et al.^10^ used the nonlinear spring hardening effect of curved springs to increase the bandwidth of an energy harvester. Conventional vibration energy harvesters with orthogonal geometries typically comprise linear resonance structures that result in narrow bandwidth, limiting their applications in real-world scenarios where the vibration spectrum is typically broadband. Li et al.^11^ reduced the initial actuation voltage of a MEMS actuator with a curved electrode exploiting the nonlinearity of electrostatic actuation; again, this cannot be achieved through orthogonal geometries. Although there are some cases where MEMS devices with complex geometries demonstrated superior performance, they have not been widely adapted to date. One reason is that they need to be designed by complex theoretical calculations. Such a design method requires considerable expertize from the designer. In addition, the design methodology is practically impossible to transfer to other devices; a case-by-case approach is required. An alternative is topology optimization, which can be used to design MEMS devices with complex geometries. Ananthasuresh et al.^12,13^ and Seshia et al.^14^ developed complex force and motion amplification mechanisms to increase the sensitivity of accelerometers. Sandia National Laboratories^15^ developed a complex motion amplification mechanism to improve the actuation displacement of an intricate micromechanical actuator in a mechanical weapon-lock system. However, these MEMS devices typically comprise simple beam (or truss) elements as a fundamental building block to form optimized topologies. However, such a methodology easily leads to designs that often cannot be fabricated, as it is difficult to incorporate fabrication constraints in the topology optimization process^14^. Another approach is to use parametric model order reduction (PMOR) to design MEMS devices^16–19^. This approach is based on a simplified model, i.e., a reduced-order model (ROM) of the mechanical structure with only a few key parameters. This limits the design space and does not allow the exploration of unconventional freeform geometries. The ROM is generated to shorten the computational time for transient and harmonic analysis at the system level. However, PMOR techniques have hardly been exploited to date for systematic optimization with an evolutionary algorithm.

Therefore, although there have been some examples of MEMS devices with complex geometries that demonstrated superior performance, there is a lack of a simple design methodology for designing MEMS devices with freeform geometries. This paper describes a novel, semiautomated design methodology for a wide range of MEMS devices allowing freeform geometries. The proposed method can design and optimize MEMS devices for different applications that can be fabricated and are robust to fabrication tolerances. Furthermore, it does not require much computation time and memory resources. The use of freeform geometries allows higher degrees of freedom in the design process, improving the diversity and potentially the performance of the MEMS devices.

**Fig. 1:**
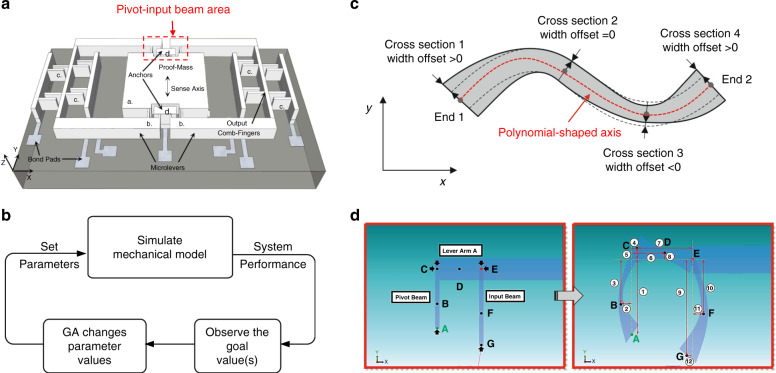
Optimization process and full parameterized AMPACC model and freeform geometries. **a** Schematic view of the parameterized AMPACC model comprising a **a** proof mass, **b** microlevers, **c** output comb fingers, and **d** anchors. The direction of motion is along the *y*-axis. **b** Generic process flow of the proposed optimization system. **c** Freeform beam model in MEMS+^22^. **d** The orthogonal design of the pivot-input beam from ref. ^20^ is changed into freeform beam structures defined by parameters noted in red and blue in Table 1

The novel design approach is demonstrated for a MEMS accelerometer comprising a mechanical motion preamplifier^20,21^. A schematic drawing of the accelerometer (termed AMPACC in the following) is shown in Fig. 1a. It features a proof mass (a), amplification microlevers (b), and differential comb fingers at the end of the microlevers for capacitive readout (c). When the sensor is subjected to an acceleration along its sensitive axis (*y*-axis in Fig. 1a), the proof mass moves along the same axis. This displacement is mechanically amplified and transferred to the output through the microlevers. Each pair of microlevers has its output connected to a differential comb finger stage, where the amplified motion can be measured by a differential change in capacitance. However, the potential of the approach is far from fully exploited, as a mechanical motion preamplifier is a complex compliant lever structure. The design described in ref. ^20^ was based only on simple orthogonal beam elements. In the work presented here, we replace the OD with a freeform design (FD) to further improve the performance of the mechanical amplifier, resulting in an accelerometer of better performance.

The paper is organized as follows. After the introduction, Section “Results” describes the general design and optimization methodology for compliant structures with a case study of the optimization of an AMPACC, as well as the experimental result. Section “Discussion” describes the advantages introduced by applying freeform geometries to an AMPACC and the convergence of the optimization. Section “Materials and methods” presents the fabrication process used and the experimental setup. Section “Conclusion” concludes the paper.

## Result

### Optimization system based on a genetic algorithm (GA)

The proposed design methodology comprises two parts: (i) a parametrized mechanical finite element model implemented with Coventor MEMS+^®^^22^ and (ii) a GA implemented with MATLAB^23^; the latter is illustrated by the flow chart in Fig. 1b. First, designers must build a fully parameterized mechanical model in MEMS+ representing the device to be optimized. This follows a modular approach by simply selecting and connecting pretested, full parameterized components in an intuitive 3D graphical interface. To enable freeform geometries, MEMS+ provides a freeform beam component, as illustrated in Fig. 1c. Each component has an underlying behavioral model, which is accessible via the MATLAB scripting interface. Thus, with the GA toolbox in MATLAB, a design process can be developed based on a GA that optimizes the MEMS+ model by varying the parameter values of each component. It should be noted that the parameter values during optimization need to be as dispersed as possible within the parameter space to guarantee a global rather than a local optimal solution. As a FD has a vast number of degrees of freedom, exploring the parameter space by a simple nested sweep of all parameters is computationally very intensive. The GA helps to solve this problem, as will be explained later; however, it is nevertheless important for the designer to balance the computation cost and parameter scanning range. Details of each step of the proposed optimization algorithm are discussed in the following.

A GA is based on the principles of natural selection and genetics, combining the fittest individuals in the population to search for the best solution. These evolutionary-based techniques are excellent for complex optimization problems, for which they can find optimal solutions in a short period of time. For optimization, the GA sets the parameter values of the mechanical model and simulates each “individual” set in the first generation. Using a figure of merit (FOM, discussed in section “Results”) as a performance goal function, the GA generates a new parameter set for the next generation. After several generations, the parameter values converge, indicating that the mechanical model has reached an optimal design.

The mechanical finite element model (at least the parts to be optimized) must be completely parameterized. This means that all relevant geometric parameters must be represented by variables rather than set as fixed values. In addition, a range and associated constraints of all mechanical parameters must be specified. For the AMPACC investigated here, 34 parameters listed in Table 1 (and illustrated in Fig. 1a) were used to define the shape, and they specify dimension boundaries for the proof mass, microlevers, output rotors, fingers, and so on.

**Table 1:**
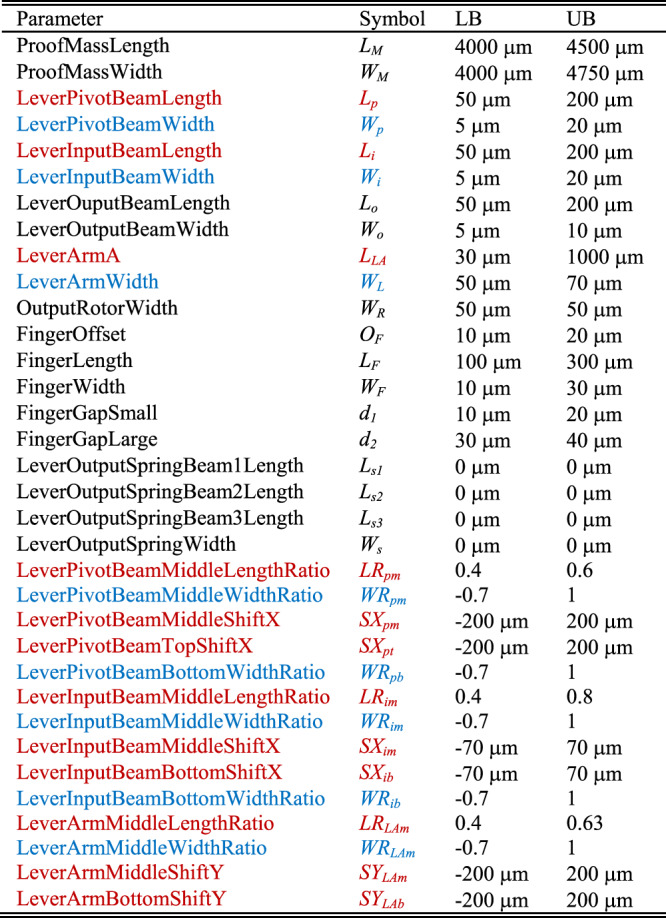
Definition, symbol, and upper, and lower bounds of 34 parameters

Parameters noted in black define the parts of the AMPACC except the “pivot-input beam area” as defined in Fig. 1a

Parameters noted in red and blue define the “pivot-input beam area” in an AMPACC, as shown in Fig. 1d. Parameters noted in red define the coordinates of the six cross-section points B to G along the polynomial-shaped axes in Fig. 1d. Parameters noted in blue define the cross-section widths of the seven cross-section points A to G in Fig. 1d

In particular, the crucial part of the AMPACC design is the “pivot-input beam area” indicated by the red box in Fig. 1a; this geometrical part affects not only the overall stiffness of the suspension system but also the amplification ratio^20^. An increase in the pivot width leads to a higher amplification ratio of the lever, which increases the output deflection at the end of the microlevers. Increasing the pivot width also makes the overall suspension of the device stiffer and therefore reduces the output deflection (i.e., sensitivity) at the end of the microlevers. These two effects form a tradeoff between the amplification ratio and the overall suspension stiffness, determining the sensitivity of the accelerometer. Since AMPACC relies on a complex compliant lever mechanism, the original ODs presented in ref. ^20^ were based only on simple orthogonal beam structures. However, such designs do not fully explore the potential of the AMPACC. More complex, freeform geometrical shapes may result in superior performance. Thus, we propose to replace the simple orthogonal structures with freeform structures, which may offer a solution to overcome the dilemma of contradicting trends for stiffness and displacement, as mentioned above.

However, such a FD requires substantially higher degrees of freedom to control the shapes of the different geometries. In addition, the complex mechanisms and nonlinearities of freeform geometries make it difficult to analytically design them, let alone systematically optimize them. With the help of commercial simulation software packages, such as COMSOL^24^, MEMS+ (from Coventor^22^), or ANSYS^25^, this aspect can be approached by finite element analysis (FEM) simulations. In this paper, MEMS+ was chosen to simulate freeform geometries due to its compatibility with MATLAB, low computational resource requirements and the capability for cosimulation with Simulink. Most importantly, the freeform geometries can be defined by Bezier curves, allowing us to describe a curve by three points; this minimizes the number of optimization parameters. In MEMS+, freeform geometries can be formed by simply varying the widths and coordinates of cross-sections at different locations along a polynomial-shaped axis, as illustrated in Fig. 1c^22^.

Figure 1d shows how the OD of a pivot-input beam is changed into a freeform beam structure defined by cross-section widths and the (*x*, *y*) coordinates of several points. The coordinates of the six cross-section locations B to G along the polynomial-shaped axes are defined by parameters noted in red in Table 1. The coordinates of the cross-section at location A are determined by the proof mass width and length. The relation among different parameters is explained in Fig. 1d and Table 2. The cross-section widths of the seven cross-section locations A to G are defined by parameters noted in blue in Table 1 and defined in Fig. 1d and Table 3. Therefore, this set of variables fully describes the device geometry and the FD of the pivot-input beam, given that the anchor sizes and gaps are assumed to be constant, based on fabrication rules.

**Table 2:** Definition of the 12 dimensions shown in Fig. 1d

Label in Fig. 1d	Definition	Note in Fig. 1d	Definition
①	*L*_*p*_	⑦	*L*_*LA*_
②	*SX*_*pm*_	⑧	*SY*_*LAm*_
③	*LR*_*pm*_ * *L*_*p*_	⑨	*L*_*i*_
④	*SX*_*pt*_	⑩	*LR*_im_* L_*i*_
⑤	*SY*_*LAb*_	⑪	*SX*_*im*_
⑥	*LR*_LAm_* L_*LA*_	⑫	*SX*_*ib*_

**Table 3:** Gaps between elements in the design and anchor sizes are assumed to be constants for the optimization process

Cross-section point	A	B	C	D
Cross-section width	(1 + *WR*_*pb*_)*W_*p*_	(1 + *WR*_*pm*_)*W_*p*_	*W*_*L*_	(1 + *WR*_*LAm*_)*W_*L*_
Cross-section point	E	F	G	
Cross-section width	*W*_*L*_	(1 + *WR*_*im*_)*W_*i*_	(1 + *WRib*)*W_*i*_	

The 34 parameters shown in Table 1 are defined with lower and upper bounds (LBs and UBs, respectively) determined either (i) by fabrication rules or (ii) by a qualified guess by the designer of the optimum value.

According to Table 1, the upper bounds of the proof mass dimensions are determined by the fabrication process described in ref. ^26^, as are the lower bounds of the beam and finger widths as well as the width of the output spring. The lower bound of the finger gap size is the minimum feature size of 2 µm (again determined by fabrication rules). The upper bound of the lever arm width is 70 µm, which is set to make a release etch possible (approximately 30 µm under cutting from both sides was assumed to be feasible^20^). The output rotor width in Fig. 1a is allowed to be larger than the release etch dimension, as the area under the output rotor is opened up from the backside of the wafer. This is done to avoid out-of-plane motions of the output rotors that may cause stiction between the rotors and the substrate. The lower bound of the length of lever arm A is set to 30 µm to ensure that the gap between the microlever anchor and the proof mass is bridged. The upper bound of the finger gap is also limited by the fabrication process^26^. The other bounds are chosen by what is intuitively thought to be a range of potentially optimal solutions, based on the OD described in ref. ^20^. As the LBs and UBs are used for the GA optimization, setting the parameter range too large slows down the algorithm, but setting the range too small risks missing the optimum.

Further to the parameterization of the design, a set of geometrical design constraints must be defined due to fabrication limits^26^. Seven constraints are suggested after inspecting the design and are listed in Table 4.

**Table 4:** Constraints imposed by the geometry on the design parameters

1	*L*_*LA*_ > *L*_*ac*_*/2* *−* *O*_*p*_ + *W*_*i*_*/2*
2	*L*_*LA*_ < *L*_*M*_*/2* *−* *O*_*p*_ − *W*_*i*_*/2*
3	*L*_*s1*_ < *L*_*F +*_ *O*_*F*_ − *W*_*R*_*/2* + *W*_*Sa*_ − *W*_*s*_*/2*
4	*L*_*s2*_ > 2*(W_*s*_*/2)* *+* *g*_*ML*_
5	*L*_*s3*_ < *L*_*s1*_ + *W*_*o*_*/*2 + *W*_*R*_*/2* + *L*_*F*_ + *O*_*F*_ + *W*_*Sa*_ + *g*_*MS*_ + *L*_*M*_*/*2
6	*W*_*s*_ < *W*_*L*_
7	*d*_*2*_ > 2*d_*1*_

For a MEMS accelerometer, the sensitivity versus bandwidth is often a tradeoff. Ideally, high sensitivity and large bandwidth are desired. Thus, the product of the sensitivity and bandwidth (SBWP) is included in the FOM in the following.

In addition, one of the most important limiting factors, which can also serve as a good comparison metric between devices, is the footprint. A larger footprint generally allows for a better device. Thus, we also include the chip size (area) in the FOM to compare vastly different devices under the same common restriction.

Moreover, the lowest spurious out-of-plane mode (at frequency *f*_su_) should not be close to the sensing mode (at frequency *f*_se_) to prevent interference from out-of-plane movement^20^. Therefore, the GA should evolve a design alleviating this problem. We also included the ratio between the first spurious mode and the sensing mode in our FOM.

Thus, an FOM is suggested to include three factors: (i) SBWPs, (ii) die area, and (iii) frequency ratio between the first spurious mode *f*_su_ and sensing mode *f*_se_. Therefore, the FOM can be expressed as

1 $${\rm{FOM}} = {\rm{SBWP}} \times \frac{1}{{\rm{area}}} \times \frac{{f_{\rm{su}}}}{{f_{\rm{se}}}}.$$

The GA then optimizes different parameter sets to maximize the FOM.

It should be noted that in this work, both a FD and a OD are implemented, having the same die area for a fair comparison. Thus, the area value can be set to unity.

### Optimization process

In the first step of the optimization process, the GA runs 40 “individuals” (i.e., designs with a specific parameter set), which are chosen randomly within the boundaries of the parameters listed in Table 1. For each individual, an FEM simulation is carried out for the fully parameterized mechanical model. The simulation includes eigenfrequency simulation and static displacement simulation under 1 g acceleration. The simulation result is sent back to the GA in MATLAB to calculate the FOM. This calculation is performed by an objective function, which computes the FOM of Eq. (1). The parameter values of the first generation are generated by the GA randomly in the allowed range of each parameter. Once the first generation has been simulated, the GA sorts the results and then performs several postprocessing steps, including picking the five best individuals (elite preservation), deriving a certain number of new random individuals (mutation) and cross fertilizing good individuals to create offspring. This last step involves taking different parameters from different good individuals and combining them to create a new individual (child). These three steps create parameter sets for the second generation of the optimization process. Then, the GA carries out the same simulation process for the second generation as was done for the first generation.

**Fig. 2:**
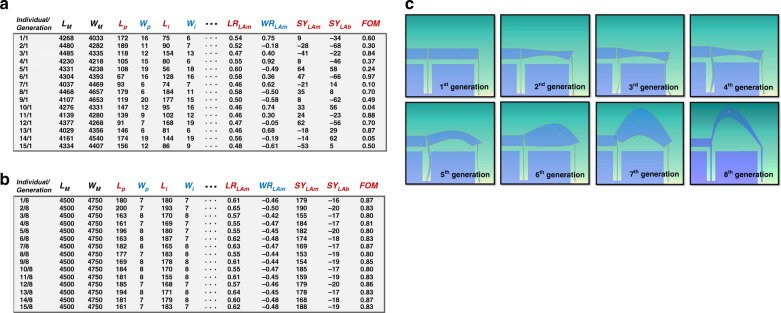
Optimization process. **a** Example individual in the optimization process: 1st–15th individual of the 1st generation (1/1 to 15/1). **b** Example individuals in the optimization process: 1st to 15th individuals of the 8th generation (1/8 to 15/8). **c** The shape of pivot-input beams changes during optimization

For each simulation, a row of values is recorded. Figure 2a shows the results from the 1st to the 15th individual of the 1st generation (1/1 to 15/1). The FOM varies considerably, indicating that the system still explores the design space. The GA works toward systems that have a large FOM. Figure 2b shows the 1st to the 15th individual of the 8th generation (1/8 to 15/8). The GA consistently settles towards designs with higher FOMs. After several generations, the GA starts to converge. Figure 2c shows the changing shape of the parameterized mechanical model during the optimization process.

The next step in the design process is a robustness analysis, which starts by collecting 20 designs with the highest FOM and making them optimal design candidates.

For the robustness analysis, another user-defined parameter determines the number of Monte Carlo simulations to be performed for each individual optimal design candidate; here, this parameter was chosen to be 100. Monte Carlo simulations were then performed on each remaining individual using user-specified standard deviation values for all parameters. This represents the fabrication tolerances. The robustness analysis can vary more design parameters than the GA. For example, parameters such as ProofMassLength and ProofMassWidth were varied for the robustness analysis by 2%. This reflects the typical fabrication tolerances for a micromachined sensing element^26^. One-hundred Gaussian distributed parameter sets were calculated for all parameters of an individual using their mean values and user supplied standard deviations. Therefore, for each individual, 100 simulations were run, and the FOMs were recorded. A yield value was calculated, representing the percentage of the simulations for each individual above the minimum FOM. The user finally chooses one as the final design by reviewing the yield and performance of the investigated individuals.

### Optimization results

**Fig. 3:**
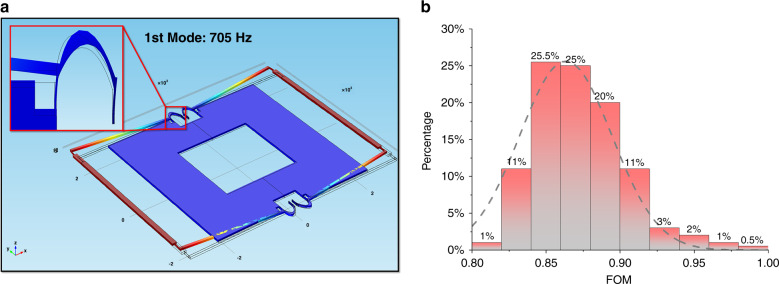
Optimization results. **a** FEM simulation of the first mode of the optimal FD. **b** Distribution of the FOM in the Monte Carlo simulations of the optimal FD

For the designs presented in this work, the GA optimization process ran continuously for 8 generations, with each generation having a size of 40 individuals. The optimal FD emerged with an FOM of 0.860. It had a resonant frequency of 705 Hz, as shown in Fig. 3a, and a sensitivity of 1.01 μm/g. The frequency ratio between the first spurious mode and sensing mode was 1.208. The optimal values of different parameters are listed in Table 5.

**Table 5:**
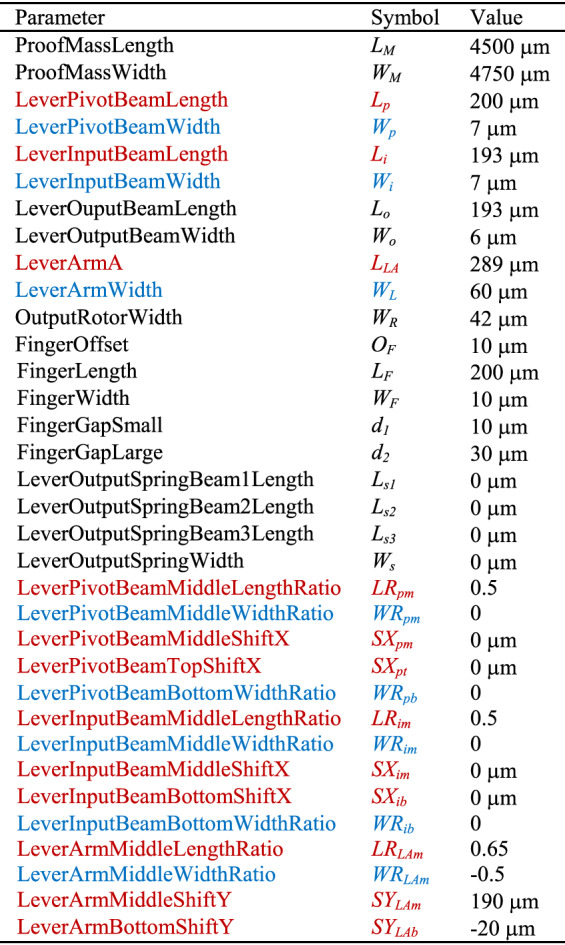
Parameter value of the optimal AMPACC with freeform geometries

For a fair comparison, an optimization was also run for an OD, resulting in a design with an FOM of 0.457. It had a resonant frequency of 943 Hz and a sensitivity of 0.412 μm/g. The frequency ratio between the first spurious mode and sensing mode was 1.174. By comparing the two designs, the FOM and sensitivity were improved by 88% and 145%, respectively.

The results of the robustness analysis of the FD are shown in Fig. 3b; it had a yield of 62.5% with a minimum FOM of 0.86.

Table 6 compares the optimal FD with other designs. They include the initial design (ID), an optimal OD, a conventional MEMS accelerometer design from ref. ^26^ (CD), and the design by Zeimpekis et al.^20^ (ZD).

**Table 6:** Comparison of different designs

Property	FD	ID	OD	CD	ZD	IFI (%)	IFD (%)	IFC (%)	IFZ (%)
Output displacement (μm)	1.01	0.149	0.412	0.273	0.300	578	145	270	237
Sensing mode (kHz)	0.705	1.624	0.943	0.952	1.007	−57	−25	−26	−30
SBWP	0.712	0.245	0.389	0.260	0.302	191	83	174	136
Chip size (mm^2^)	39.3	39.3	39.3	39.3	39.3	0	0	0	0
Spurious mode (Hz)	0.852	1.807	1.108	3.754	1.159	−53	−23	−77	−26
*f*_su_/*f*_se_	1.208	1.113	1.174	3.943	1.151	9	3	−69	5
FOM	0.860	0.273	0.457	1.025	0.348	215	88	−16	147
Proof mass displacement (μm)	0.096	0.009	0.014	0.273	0.010	967	586	−65	860
Amplification ratio	10.5	16.5	29.4	1.0	30.0	−36	−64	950	−65
Proof mass (mg)	2.125	2.125	2.125	1.858	1.205	0	0	14	76

*FD* optimal freeform design, *ID* initial design given to the optimization process, *OD* optimal orthogonal design, *CD* conventional MEMS accelerometer design^26^ scaled up to the same chip size as ZD: Zeimpekis et al. design^20^ scaled down to the same chip size as FD

IFI = (FD − ID)/ID; IFD = (FD − OD)/OD; IFC = (FD − CD)/CD; IFZ = (FD − ZD)/ZD

ID is provided as input to the optimization process. CD and ZD were scaled up to the same chip size of the FD. The minimum width of the suspension system in all designs was set to 10 μm. In Table 6, columns IFI, IFD, IFC, and IFZ show the improvements of FD compared with ID, OD, CD, and ZD, respectively.

The output displacement was influenced by both the amplification ratio and the proof mass displacement. In terms of amplification ratio, FD showed a decrease of 64% and 65% compared with OD and ZD, respectively. However, in terms of the proof mass displacement, FD showed an increase of 586% and 860% compared with OD and ZD, respectively. Thus, the output displacement of FD indicated an increase of 145% and 237% compared with that of OD and ZD, respectively. The resonant frequency of FD was 25% and 30% smaller than that of OD and ZD, respectively. Finally, in terms of SBWP, FD showed an increase of 83% and 136% compared with OD and ZD, respectively.

Moreover, FD indicated increases of 3% and 5% compared with OD and ZD, respectively, in terms of the ratio between the first spurious mode and the sensing mode (i.e., *f*_su_/*f*_se_).

Finally, the FOM of FD was 1.88 and 2.47 times that of OD and ZD, respectively.

It should be noted that all designs compared in Table 6 had the same die area. In terms of sensitivity, all AMPACC designs (FD, OD, and ZD) showed an increase (270%, 51% and 10%, respectively) compared with the CD. Especially in terms of SBWP (the product of sensitivity and bandwidth), FD, OD, and ZD showed increases of 174%, 50%, and 16%, respectively, compared with CD. Therefore, there is clear evidence that mechanical amplification improved the MEMS accelerometer in terms of the product of sensitivity and bandwidth, which are the two most important parameters for a MEMS accelerometer. However, in this paper, apart from SBWP, we also took the difference between the spurious and fundamental modes, i.e., the ratio *f*_su_/*f*_se_, into consideration. Considering only this ratio (*f*_su_/*f*_se_), FD, OD, and ZD showed decreases of 69%, 70%, and 71%, respectively, compared with CD. This is the reason why in terms of the final FOM, CD had the highest value. However, it does not indicate that mechanical amplification is unbeneficial for improving the performance of MEMS devices.

Figure 2c shows a graphical illustration of the optimization process of the pivot arm. The GA changed the shape of arm A considerably. During the optimization, the GA attempted to make the pivot-input area less stiff, which reduced the total stiffness of the accelerometer and increased the proof mass displacement. However, a less stiff pivot-input area decreased the amplification ratio and the resonant frequency, which are undesired results. Thus, the GA attempted to find a compromise between the two trends to reach a high SBWP by increasing the output displacement of the lever and keeping the resonant frequency as little influenced as possible. Finally, the GA reached a solution whose output displacement is increased by 578%, while the resonant frequency was reduced by only 57% compared with that of the ID, as indicated in Table 6.

It is worth mentioning that the whole optimization process took 12 h with a 3D mechanical model and 8 h with a 2D mechanical model by using a laptop with an i7 core running at a 2.5 GHz working frequency and 8 GB RAM.

### Experimental results

**Fig. 4:**
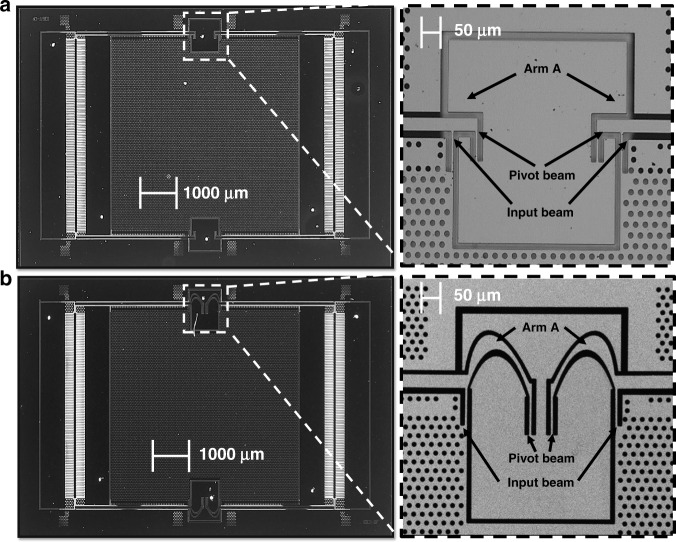
Fabrication results. **a** SEM image (top left) of the chip (top right) and pivot-input beams of the optimized orthogonal design: AMPACC with orthogonal “pivot-input beam area”. **b** SEM image (bottom left) of the chip (bottom right) and pivot-input beams of the optimized freeform design: AMPACC with freeform “pivot-input beam area”

**Fig. 5:**
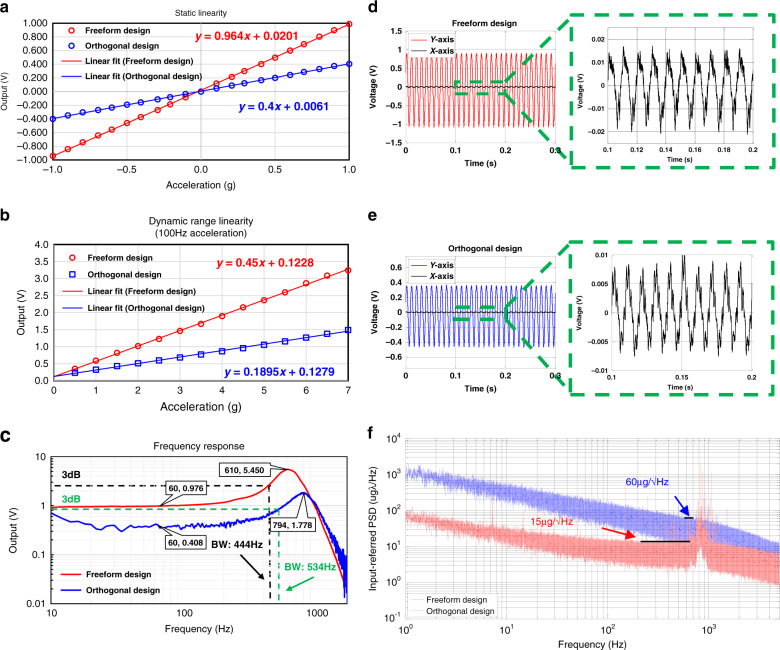
Experimental tests. **a** Output signals of the FD and OD for a range of ±1g. The linear fits were plotted using the least squares method. The nonlinearities of both designs were 3% in the worst case. **b** Dynamic range linearity measurements for the FD and OD using a mechanical shaker that varied the acceleration amplitude from 0.5 to 7g at 100Hz with a step of 0.5g. The results indicate a nonlinearity of 3% up to approximately ±7g. **c** Frequency responses of the FD and OD. **d** Cross-axis sensitivities of the two designs under 1g acceleration at 100Hz. The cross-axis sensitivity of the FD is 1.350% (0.960V/g in the *y*-axis, 0.013V/g in the *x*-axis). **e** Cross-axis sensitivities of the two designs under 1g acceleration at 100Hz. The cross-axis sensitivity of the OD is 1.500% (0.400V/g in the *y*-axis, 0.006V/g in the *x*-axis). **f** Input-referred noise floor PSD of the FD and OD

Figure 4a, b shows the layout and the fabricated structure, which match well. The linearity of the FD and OD was evaluated under static and dynamic accelerations. Figure 5a shows a static linearity comparison between the FD and OD for a range of ±1 g. According to the linear fit equations shown in Fig. 5a, the FD featured a sensitivity (scale factor) of 0.964 V/g, while the OD had a sensitivity of 0.400 V/g. The nonlinearity was calculated as the maximum deviation of the mechanically amplified output voltage from the best fit line as a percentage of the full scale of measurements (−1 to 1 g). The maximum nonlinearities of both designs were 3% for the amplified output within this range. Thus, the curved beams did not influence the static linearity of the AMPACC.

To compare the linear open-loop response and the dynamic range of the FD and OD, the two designs were evaluated on a mechanical shaker setup for accelerations ranging from 0.5 to 7 g at 100 Hz with a step of 0.5 g.

As shown in Fig. 5b, for both designs, the full-scale maximum nonlinearity measured at the full-scale dynamic acceleration of ±7 g was 3%. The maximum sensor deviation of the sensor output from the linear best fit line occurred when the acceleration reached ±7 g. Thus, the curved beams also did not influence the linearity of the open-loop dynamic range of the AMPACC.

The frequency response measurements were repeated on the shaker system. As shown in Fig. 5c, the natural frequency of the FD was 610 Hz, while that of the OD was 794 Hz. The measured resonant frequencies of both designs were 13–16% lower than the simulated values, which is likely due to overetching of the structures during fabrication. However, the ratio between the measured resonant frequencies of the FD and the OD was 0.77, which is close to the simulated value of 0.75. The bandwidth of the FD was 444 Hz, while the bandwidth of the OD was 534 Hz.

To compare the cross-axis sensitivities of the FD and OD, measurements were carried out on the shaker setup for 1 g acceleration at 100 Hz along the *y*- and *x*-axes shown in Fig. 1a, respectively (*y* being the sense axis). The cross-axis sensitivity is the output voltage under an acceleration along the *x*-axis as a percentage of the output voltage under the same acceleration along the *y*-axis. Figure 5d, e shows that the cross-axis sensitivities of the FD and OD were 1.35% (0.96 V/g in the *y*-axis, 0.013 V/g in the *x*-axis) and 1.5% (0.40 V/g in the *y*-axis, 0.006 V/g in the *x*-axis), respectively, which are quite close. Thus, the curved beams did not influence the cross-axis sensitivity of the AMPACC.

Figure 5f shows the comparison of the FD and OD in terms of the input-referred noise floor power spectrum density (PSD). Most of the spikes in the PSD are due to power supply frequency noise, as they were integer multiples of 50 Hz; this can be alleviated by better shielding. The input-referred noise floor of the FD was 15 µg/√Hz in a frequency range between 100 and 650 Hz, while that of the OD at 400 Hz was 60 µg/√Hz. Thus, a significant improvement in resolution by a factor of approximately four was obtained using the freeform geometries, demonstrating the improvement in performance that is achievable by employing this new design method. The noise floor of both designs was nevertheless rather high compared to state-of the art. This is attributed mainly to the electronic noise of the pickoff circuit, as a simple printed circuit board with discrete components was used.

## Discussion

### Comparison of different designs

The proposed design methodology greatly expedites the design process and gives much greater confidence that the results are both optimal and robust against fabrication tolerances. Without robustness analysis, it is possible to design a system that may easily become unfeasible to fabricate. We experimentally verified the effectiveness of this design approach by optimizing a MEMS accelerometer comprising a mechanical motion amplifier. Experimental results show an SBWP improvement of 100% and a sensitivity improvement of 141%, as indicated in Table 7. It should be noted that we took the resonant frequency rather than the −3 dB cutoff frequency when calculating the SBWP of the simulated results, which is the main reason why the SBWP of the simulated results deviates from that of the experimental results. Furthermore, the performance parameters of the accelerometer, such as the static nonlinearity, linear open-loop dynamic range and cross-axis sensitivity, was not influenced by the introduction of freeform geometries. Thus, the novel design methodology efficiently designed a freeform structure superior to a conventional OD.

**Table 7:** Comparison between the simulated and experimental result of the optimal OD and FD

	Sensitivity	Bandwidth	SWBP
*Experiment*			
OD	0.400 V/g	0.534 kHz	0.214
FD	0.964 V/g	0.444 kHz	0.428
Relative change	141%	−17%	100%

	Sensitivity	Resonant frequency	SWBP
*Simulation*			
OD	0.412 μm/g	0.943 kHz	0.389
FD	1.010 μm/g	0.705 kHz	0.712
Relative change	145%	−25%	83%

### Convergence of optimization

**Fig. 6:**
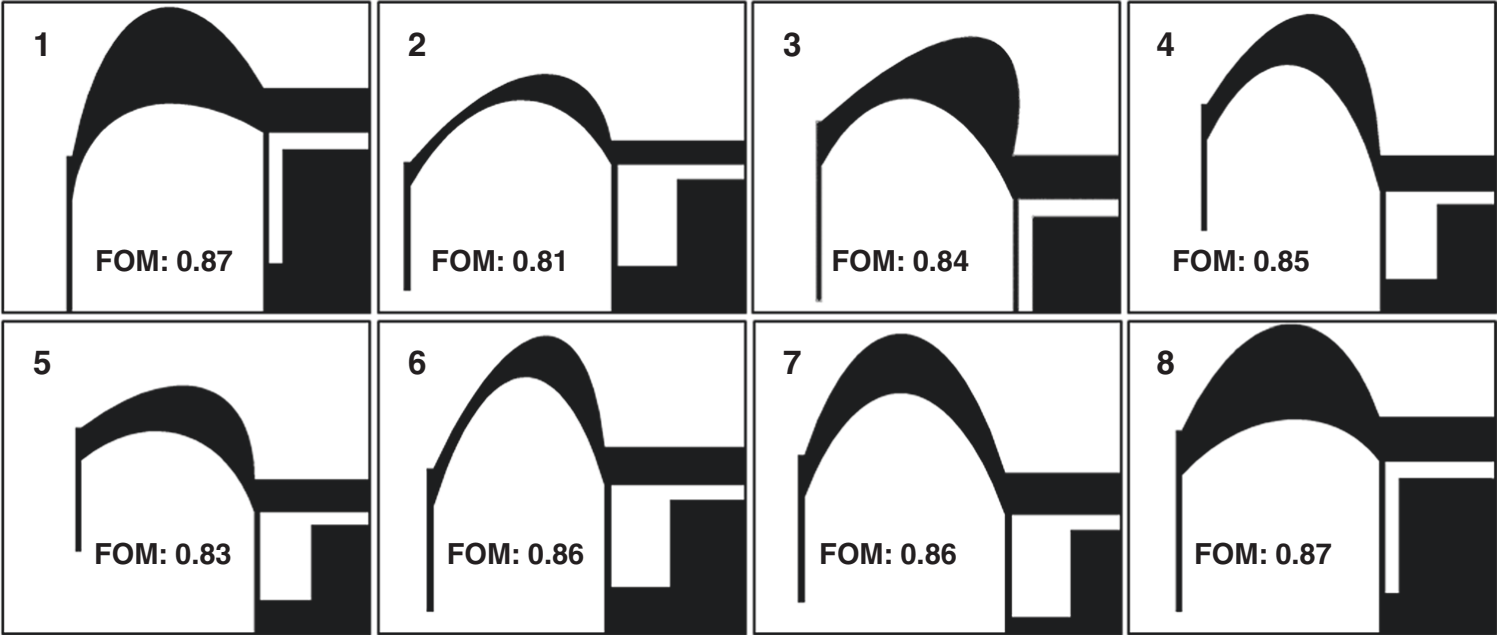
Optimal shapes of the pivot-input beam area in eight different GA optimization runs with different initial conditions

The parameter values during optimization need to be as dispersed as possible within the optimum parameter space to guarantee a global rather than a local optimal solution. This is potentially the greatest limitation of the method presented here as a FD with a vast number of degrees of freedom; it scales the local minima in a nonlinear fashion and therefore becomes heavily computationally intensive. To minimize this effect, the degrees of freedom were relatively reduced in this study. As circumstantial evidence, eight independent optimization processes with different initial conditions spanning the design space were carried out. The solutions achieved by the GA optimization process are shown in Fig. 6. The resulting topologies in the eight cases are rather similar, suggesting a global convergence of the optimization process to a large extent. The final FOMs of the eight different optimization processes are in the range between 0.81 and 0.87.

In conclusion, further investigation is still needed to fully verify the convergence of the optimization process. This includes (i) increasing the diversity of the initial shapes to cover more possibilities and investigating the influence of an initial shape and (ii) using different optimization algorithms, such as particle swarm optimization^27^ and the simulated annealing (SA) algorithm^28^, to investigate the influence of the algorithm itself on the optimal solution.

## Conclusion

The presented work introduced a novel and powerful design methodology that can be applied to a wide range of MEMS devices relying on compliant mechanisms. The GA-based algorithm running within MEMS+ is capable of quickly and efficiently designing and optimizing high-performance compliant mechanisms for many MEMS devices. A MEMS accelerometer comprising a mechanical motion amplifier was designed by this method. Experimental results indicate an improvement in the product of sensitivity and bandwidth by 100% and a sensitivity improvement by 141% compared to a device designed with conventional orthogonal shapes.

SA was also used to optimize an AMPACC with freeform geometries, which was taken as a reference to compare the proposed GA design method. The SA algorithm is inspired by the physical process of heating a metal beyond the melting point and then gradually lowering the temperature to end up with a solid with minimum structural defects^29,30^. SA can be adopted for the design optimization of MEMS devices^31–33^. A similar approach was used for the design of an AMPACC. The optimal solution of the SA after 14 h of computation had a resonant frequency of 0.827 kHz, a displacement of 0.334 μm under 1 g acceleration and a *f*_su_/*f*_se_ of 1.112. Its SWBP was 0.276, and the overall FOM was 0.307. The time for the SA to reach the optimal solution was comparable to the proposed GA design method. However, in terms of SWBP and FOM, the optimal design of the SA decreased by 61% and 62%, respectively, compared with that of the GA (using design FD as a reference). In addition, the optimal solution of the SA depended more on the initial parameter value set than the GA-based method, as the FOM varied from 0.130 to 0.307 in eight different optimization process runs. On the other hand, the GA started with a widely dispersed set of values for all parameters at the beginning of the optimization. The FOM of different optimization process runs converged to a large extent, as shown in Fig. 6.

The presented methodology can be applied to many other MEMS devices comprising freeform geometries and is expected to result in rather unusual geometrical shapes unseen in MEMS designs to date. It is worth mentioning that apart from the MEMS accelerometer discussed in this work, we also have optimized a MEMS actuator, specifically a MEMS microgripper, by the proposed methodology. The overall objective was to achieve a large displacement with a small actuation voltage. The performance of the microgripper was improved significantly with the introduction of freeform beams, as they can achieve a lower spring constant compared with conventional orthogonal beams. Our preliminary study showed that for the same actuation voltage, a microgripper with freeform geometries improved the displacement by 150–200% compared with orthogonal geometries in the same die area. Therefore, freeform geometries have two advantages: (i) higher energy efficiency (lower actuation power to reach the same displacement) and (ii) less harm to fragile objects during gripping and releasing. The microgripper with freeform geometries had a large displacement (91 μm) with a low actuation voltage (47.5 V), which agreed well with the theory. This made it possible to manipulate a wide range of objects (size ranging from 10 to 100 μm). Compared with two state-of-the-art electrostatic microgrippers in the literature^34,35^ (in terms of actuation ability or range), microgrippers with freeform geometries have a larger gripping range. These results will be published in due course.

Planned future work includes further investigation of the convergence and parallel computation of this design method. In addition, the current design method includes parameters in the mechanical domain only. The inclusion of parameters from other physical domains (such as thermal and electronic domains) is expected to further demonstrate the strength and universality of this design method.

The noise floor of the designed devices was limited by the electronic interface circuit. A capacitive sensing application-specific integrated circuit with a noise floor of 50 aF/√Hz^36^ can be further used to reduce the noise floor of the designed devices and to effectively utilize the improved deflection of freeform beams. In additional future work, we plan to implement the sensor in a closed-loop control system.

## Materials and methods

### Fabrication process

**Fig. 7:**
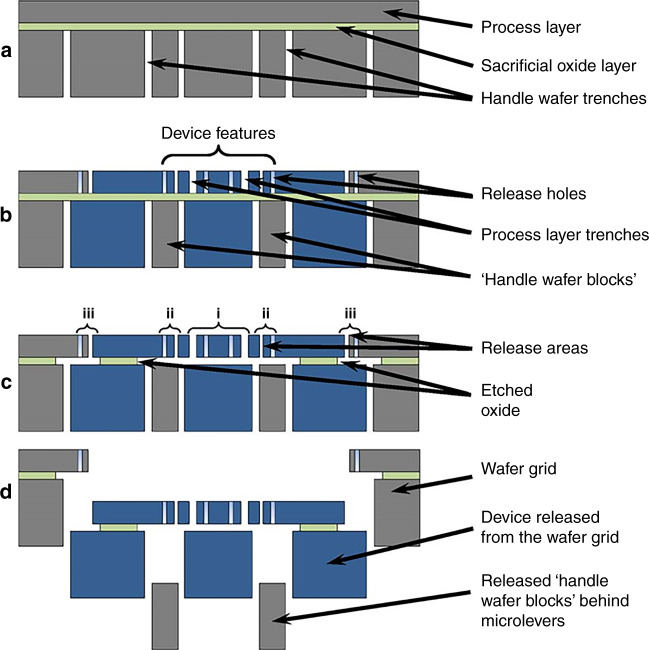
Fabrication process. Fabrication flow of the MEMS accelerometers: **a** Backside etching using DRIE to define the backside trenches. **b** Front-side DRIE to pattern the device features, release holes, and front-side trenches. **c** Three release regions, namely, the (i) device, (ii) handle wafer block release features, and (iii) dicing features, are etched consecutively by vapor phase hydrofluoric acid. **d** Device separation after release^26^

Figure 7 shows the silicon-on-insulator-based process flow used in this work, which is similar to the process described in ref. ^26^. While a pattern of frame trenches is etched on the handle layer of a wafer by deep reactive-ion etching, another pattern of trenches and etch holes is etched on the front side in a 50-μm-thick device layer. The handle layer beneath the lever and comb finger area is removed (as shown in Fig. 2) to increase the yield and reliability of the fabrication process. This is achieved by HF vapor phase etching of the BOX layer as the two trench patterns are offset by 40 μm. This allows us to separate the dies from each other without a dicing step.

We fabricated 50 chips on a 4-in. wafer, including FDs and ODs. Each chip had a size of 8 × 10 mm^2^. As listed in Table 8, 60–70% of all chips were structurally intact after release, and 40–50% were fully functional after bonding and packaging. The fabrication result indicated that the yield rate of freeform MEMS devices was good and not different from that of ODs as long as the rules concerning minimum feature size (such as minimum etching trenches and minimum widths) were adhered to.

**Table 8:** Fabrication yield

Design	Structurally intact	Good functionality
FD	15 (60%)	10 (40%)
OD	17 (68%)	12 (48%)

### Experimental setup

**Fig. 8:**
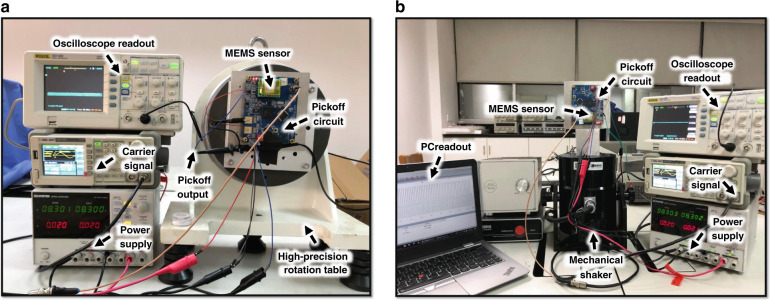
Measurement setup. **a** Measurement system for the measurement of static linearity. **b** Measurement system for the measurement of dynamic range, bandwidth, and cross-axis sensitivity

The sensitivity (scale factor), static linearity, linear open-loop dynamic range, bandwidth, and cross-axis sensitivity of the FD and OD were evaluated with an identical measurement setup, as shown in Fig. 8a, b. The two designs had sensing capacitances of the same value and were measured with the same capacitive pickoff circuit.
